# Aberrant frontolimbic circuit in female depressed adolescents with and without suicidal attempts: A resting-state functional magnetic resonance imaging study

**DOI:** 10.3389/fpsyt.2022.1007144

**Published:** 2022-10-25

**Authors:** Mengqi Liu, Yang Huang, Xuemei Li, Yang Liu, Renqiang Yu, Yicheng Long, Fajin Lv, Xinyu Zhou

**Affiliations:** ^1^Department of Radiology, The First Affiliated Hospital of Chongqing Medical University, Chongqing, China; ^2^Department of Psychiatry, The First Affiliated Hospital of Chongqing Medical University, Chongqing, China; ^3^Department of Psychiatry, The Second Xiangya Hospital, Central South University, Changsha, China; ^4^National Clinical Research Center for Mental Disorders, Changsha, China

**Keywords:** functional MRI, adolescent, female, major depressive disorder, suicidal attempt

## Abstract

**Background:**

The neurobiological basis of suicidal behaviors among female adolescents with major depressive disorder (MDD) remains largely unclear.

**Materials and methods:**

Fifty-eight drug-naïve, first-episode female adolescent MDD [including 31 patients with suicidal attempt (SA group) and 27 patients without SA (non-SA group)], and 36 matched healthy controls (HCs) participated in the present study. Resting-state functional magnetic resonance imaging (MRI) was performed on each subject. The metrics of the amplitude of low-frequency fluctuation (ALFF), fractional ALFF (fALFF), and regional homogeneity (ReHo) were compared among the three groups. Then seed-based functional connectivity (FC) was conducted based on the ALFF/fALFF and ReHo results, which were then correlated to clinical variables.

**Results:**

Compared with the non-SA group, the SA group exhibited increased fALFF in the bilateral insula and right precentral gyrus, and enhanced ReHo in the left superior temporal gyrus, left middle cingulate cortex, right insula, and right precentral gyrus. Relative to the HCs, the SA group demonstrated additionally reduced fALFF and ReHo in the left middle frontal gyrus. Moreover, the SA group showed increased FC between the right precentral gyrus and the left middle frontal gyrus and left insula, and between the right insula and anterior/middle cingulate cortex compared to the non-SA and HC groups. In addition, the fALFF in the left middle frontal gyrus was positively correlated with the 17-item Hamilton Depression Rating Scale scores, and the values in the fALFF/ReHo in the right insula were positively correlated with the duration of MDD within the patient group.

**Conclusion:**

These findings highlight the multiple abnormalities of the frontolimbic circuit, which may enhance our understanding of the neurobiological basis underlying female MDD with SA during adolescence.

## Introduction

Major depressive disorder (MDD) is one of the most common mental disorders, affecting over 320 million people worldwide (1), with the burden of disease being particularly high in adolescents and young adults (2). Notably, compared to adults, adolescents with MDD are more likely to suffer recurrent depressive episodes, suicidal thoughts, and other psychiatric comorbidities (3, 4), suggesting neurobiological underpinnings between adolescent and adult MDD may differ. Additionally, suicide is a crucial public health problem and a leading cause of death among adolescents and young adults in China (5). A previous study reported that more than 25% of adolescents with MDD had a lifetime history of suicide attempt (SA) (6). These facts highlight the need for a better understanding of the neurobiological basis of adolescent MDD associated with suicidal behavior to improve clinical diagnosis and treatment strategies.

In recent years, neuroimaging technical, especially magnetic resonance imaging (MRI), has been increasingly used to study various mental illnesses. Convergent evidence from MRI studies demonstrated structural and functional abnormalities in a variety of brain regions within adolescent MDD patients. Shen et al. analyzed multiple cortical structural indices of 8,634 adolescents with FreeSurfer software and found significantly decreased global cortical metrics in frontal, limbic, and temporal areas within depressed adolescents (7). On the other hand, numerous neuroimaging studies applied resting-state functional MRI (rs-fMRI) to explore the baseline brain activity. The commonly used re-fMRI approaches include functional connectivity (FC), regional homogeneity (ReHo), amplitude of low-frequency fluctuation (ALFF), and fractional ALFF (fALFF). FC is used to probe function of remote neural circuits, and ReHo is described as an indicator of “local connectivity” (8). ALFF reflects the intensity of regional spontaneous neural activity (9), while fALFF may detect the neural activity with improved sensitivity (10). Several rs-fMRI studies revealed that compared with health controls (HCs), adolescents with MDD showed significantly reduced FC between limbic regions (e.g., amygdala, hippocampus, and insula) and prefrontal cortex (PFC, e.g., ventral PFC, dorsolateral PFC, and middle frontal gyrus) regions (11–13). Another study reported increased ReHo value in the right prefrontal gyrus of adolescents with MDD compared to HCs (14). However, only a few neuroimaging studies have investigated the functional changes associated with suicidality in adolescent patients with MDD. A recent study reported that compared with depressed adolescents and young adults without SA, patients with SA showed significantly increased FC between the left posterior insula and the left inferior frontal gyrus, and increased FC between the right posterior insula and the left superior frontal gyrus (15). Another FC study defined the left middle and superior gyri as seeds, and found altered FC of the prefrontal-parietal and prefrontal-anterior cingulate regions was related to SA in depressed adolescents (16). Significantly reduced ALFF and fALFF in the right precentral gyrus of patients with adolescent MDD with suicidal ideation after electroconvulsive therapy was also reported (17). Consequently, we infer that the limbic and PFC regions are associated with SA of adolescent MDD.

Additionally, epidemiological studies have shown that females are more prone to depression than males (18). Studies have demonstrated sex differences in brain morphology of patients with adult MDD (19, 20). In addition, amygdalar volumes in healthy adolescents also showed sex divergence (21); the left amygdala was smaller in girls than in boys. Dorfschmidt et al. studied 298 healthy adolescents with fMRI and found sexually divergent development of brain networks within the default mode network and limbic cortex (22). Another fMRI study also demonstrated sex differences in cerebellum activation during an affective go/no-go task in depressed adolescents (23). The above studies support considering sex differences in fMRI studies.

Thus, in the current study, we aimed to apply multiple rs-fMRI analysis methods to investigate the functional alteration associated with SA in first episode, drug-naïve female adolescent patients with MDD. We hypothesized that: (1) compared with female adolescents with MDD without SA and HC, we would observe abnormalities in ALFF/fALFF and ReHo among adolescent MDD with SA, especially within the limbic and PFC regions; (2) there would be abnormalities in FC between the clusters with significant group differences in ALFF/fALFF/ReHo and other brain regions among groups; (3) these abnormalities would be related to the course of MDD and symptom severity of the disease.

## Materials and methods

### Participants

Fifty-eight female adolescents with MDD were recruited from the Department of Psychiatry of The First Affiliated Hospital of Chongqing Medical University. All patients were diagnosed with MDD based on the Diagnostic and Statistical Manual 5 (DSM-5) Structured Clinical Interview (SCID) by two professional psychiatrists (XL and XZ). The patients were further categorized into two groups: suicide attempters (SA group, *n* = 31) with a history of at least one SA within 1 year prior to MRI scanning and non-suicide attempters (non-SA group, *n* = 27) without a lifetime history of SA. An SA referred to any activity performed with an intent to die and was identified by psychiatrists in clinical settings by reviewing their medical records. Additionally, 36 female HCs without a lifetime history of SA and group-matched for age were recruited *via* advertisements. Any HC with a first-degree relative suffering from psychiatric disorders was excluded. All MDD patients and HCs met the following inclusion criteria: (1) age between 12 and 18 years; (2) right-handed; (3) no history of psychotropic drug therapy, electroconvulsive therapy, transcranial magnetic stimulation, or psychotherapy; (4) no previous neurologic disorders and head traumas resulting in loss of consciousness; (5) no history of substance abuse or dependence, and (6) no comorbid psychosis disorders. Any participant with contraindications for MRI was also excluded. The 17-item Hamilton Depression Rating Scale (HAMD-17) and Hamilton Anxiety Scale (HAMA) were applied to assess the severity of symptoms. Clinical characteristics, including age and episode duration, were collected retrospectively. Written informed consent was obtained from all participants and their guardians. The study was conducted following the guidelines of the Helsinki Declaration and was approved by the Research Ethics Committee of The First Affiliated Hospital of Chongqing Medical University (approval ID: 2020-864).

### Magnetic resonance imaging data acquisition

All participants were scanned using a 3.0-T MRI system (Skyra, Siemens Healthcare, Erlangen, Germany) with a 32-channel head coil. Foam pads and earplugs were used to minimize head motion and reduce MRI scanner noise. Subjects were instructed to relax with their eyes closed without falling asleep. None of the subjects felt discomfort, and none reported falling asleep during the scan. Conventional axial T2-weighted images and fluid-attenuated inversion recovery (FLAIR) images with 5 mm slice thickness were acquired for lesion identification. Whole-brain rs-fMRI data were acquired using a gradient-echo echo-planar imaging sequence with the following parameters: repetition time (TR) = 2,000 ms, echo time (TE) = 30 ms, 36 axial slices, slice thickness = 3 mm without slice gap, flip angle = 90^°^, matrix size = 64 × 64, voxel size = 3.4 mm × 3.4 mm × 3 mm, field of view (FOV) = 220 mm × 220 mm. The fMRI scan lasted 8 min in total, and 240 volumes were obtained for each subject. The high-resolution structural image was acquired using a 3D volumetric magnetization prepared rapid acquisition gradient echo T1-weighted sequence. The corresponding imaging parameters were: 192 sagittal slices, slice thickness = 1 mm without gap, TR = 2000 ms, TE = 2.56 ms, flip angle = 9^°^, matrix size = 256 × 256, voxel size = 1 mm × 1 mm × 1 mm, FOV = 256 × 256 mm^2^. Finally, all images were manually reviewed by two senior neuroradiologists (ML and YH) to ensure that no lesions or artifacts were present.

### Data processing

The rs-fMRI data were preprocessed using Data Processing and Analysis for (Resting-State) Brain Imaging (DPABI V6.1^1^) toolbox (24), which was based on SPM12^2^ on the MATLAB R2018b (Mathworks, Natick, MA, USA) platform. First, the first 10 functional volumes were discarded to ensure signal stability, and the remaining 230 volumes were corrected for slice timing. Next, the images were realigned to the first volume for motion correction. The mean framewise displacement (FD) was subsequently computed as a measure of head motion. Any participant with mean FD greater than 0.2, or head motion exceeding 2.0 mm or 2.0° was further excluded. Like most rs-fMRI studies, motion scrubbing was not performed in our study. After motion correction, regression of nuisance signals was performed using linear signal drift, cerebrospinal fluid signals, white matter signals, and Friston-24 head motion parameters (25) as regressors to reduce the movement-related artifacts. Finally, the resulting functional volumes were spatially normalized to the Montreal Neurological Institute (MNI) space with the Diffeomorphic Anatomical Registration Through Exponentiated Lie Algebra (DARTEL) algorithm (26).

The rs-fMRI indices (ALFF, fALFF, ReHo, and FC) were also calculated using the DPABI toolbox. For the ALFF/fALFF calculation, the preprocessed fMRI data without the band-pass filter were converted to a frequency domain using a fast Fourier transform. Then, the square root of the power spectrum was calculated, and the ALFF value of each voxel was obtained as the average square root across 0.01–0.08 Hz (9). The fALFF value was figured out as the ratio of the power spectrum in the low-frequency band (0.01–0.08 Hz) to the entire frequency range (10). Afterward, the ReHo value was calculated based on the band-pass filtered preprocessed fMRI volumes. The ReHo analysis was performed on each subject by calculating Kendall’s coefficient concordance, which was used to measure the synchronicity of a given voxel to its adjacent 26 voxels (27). Subsequently, *z*-score maps of the ALFF/fALFF and ReHo images were obtained and spatially smoothed by a 6 mm full-width at half maximum (FWHM) Gaussian kernel for the following voxel-based statistical analysis. Finally, seed-based FC was computed based on the prior results of ALFF/fALFF and ReHo analysis. Once the brain regions with significant group differences in ALFF/fALFF and ReHo were identified, each region would be saved as a binary region-of-interest (ROI) mask. The average time series within each ROI was extracted from the filtered and smoothed preprocessed fMRI data. The Pearson correlation coefficient between the time series of each ROI and the entire brain was obtained and then transformed to z-scores using Fisher’s r-to-z transformation.

### Statistical analysis

The statistical analyses were performed with SPSS package (version 26.0; IBM, Armonk, NY, USA) for the demographic and clinical data. One-way analysis of variance (ANOVA) and the Kruskal–Wallis test were used for variables with and without a normal distribution, respectively. Multiple comparisons were performed by Bonferroni correction. *P*-value < 0.05 was regarded as statistically significant.

The DPABI toolbox was applied to the statistical analysis of resting-state fMRI data. ANOVA was used to certify the differences in fMRI indices (ALFF/fALFF, ReHo, and FC) among the three groups (SA vs. non-SA vs. HC) for within-group comparisons. Age and mean FD were used as covariates. Significance was determined with a cluster-level corrected threshold of *P* < 0.05 (cluster-forming threshold at voxel-level *P* < 0.001) using Monte Carlo simulation. Then, *post hoc* two-sample *t*-tests were performed to identify between-group differences in the above four fMRI indices within the brain regions identified by the previous ANOVA, and the significance level was set at *P* < 0.05 with Bonferroni correction.

Furthermore, linear regression analyses were carried out to explore the associations between fMRI and clinical features within the patient group. Mean ALFF/fALFF/ReHo/FC in each cluster with group differences were extracted using the RESTplus toolkit (28). Then, Pearson correlation was performed to measure the linear correlations between the fMRI indices and clinical variables (including HAMD scores and duration of MDD) in SPSS with a statistical significance of *P* < 0.05 (two-tailed).

## Results

### Demographics and clinical characteristics

Demographics and clinical characteristics are shown in Table 1. Participants in SA, non-SA, and HC groups were well matched in age and education level. No significant differences were observed in terms of smoking rate, alcohol drinking rate, or family history of psychiatric disorders. Both SA and non-SA groups had higher HAMD (*P* < 0.001) and HAMA (*P* < 0.001) scores than the HC group. However, the SA and non-SA patients did not differ in the duration of illness, HAMD, and HAMA scores.

**TABLE 1:** Demographics and clinical characteristics of the participants included in the present study.

Characteristics	SA (*n* = 31)	Non-SA (*n* = 27)	HC (*n* = 36)	*P*-value	*Post hoc* test		
					SA vs. non-SA	SA vs. HC	Non-SA vs. HC
Age (years)	16.0 (15.0–16.0)	16.0 (15.0–16.0)	15.5 (13.0–18.0)	0.954	*p* > 0.05	*p* > 0.05	*p* > 0.05
Education (years)	10.0 (9.0–10.0)	10.0 (9.0–10.0)	9.5 (7.0–12.0)	0.954	*p* > 0.05	*p* > 0.05	*p* > 0.05
Duration of illness (months)	12.0 (4.0–36.0)	12.0 (5.0–18.0)	NA	NA	0.131	NA	NA
Smoking (yes/no)	4/27	4/23	0/36	0.058	*p* > 0.05	*p* > 0.05	*p* > 0.05
Alcohol drinking (yes/no)	7/24	5/22	4/31	0.450	*p* > 0.05	*p* > 0.05	*p* > 0.05
Family history (yes/no)	3/28	1/26	0/36	0.139	*p* > 0.05	*p* > 0.05	*p* > 0.05
HAMD-17	20.0 (16.0–23.0)	16.0 (14.0–19.0)	0.0 (0.0–1.8)	**< 0.001**	0.373	**< 0.001**	**< 0.001**
HAMA	16.0 (13.0–21.0)	15.0 (10.0–20.0)	1.0 (0.0–1.0)	**< 0.001**	1.000	**< 0.001**	**< 0.001**

Data are expressed as median (interquartile range). Bold values indicate *p* < 0.05. SA, suicide attempters; non-SA, non-suicide attempters; HC, healthy controls; HAMD-17, 17-item Hamilton Depression Rating Scale; HAMA, Hamilton Anxiety Scale; NA, not applicable.

### Amplitude of low-frequency fluctuation, fractional ALFF, and regional homogeneity analysis

**FIGURE 1:**
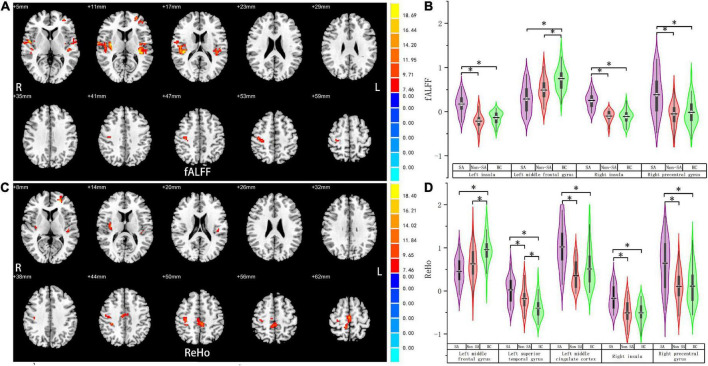
Fractional amplitude of low-frequency fluctuation and regional homogeneity analysis. Brain regions where fALFF **(A)** and ReHo **(C)** values differed significantly among SA, non-SA, and HC groups, Monte Carlo simulation corrected. *Post hoc* two-sample *t*-tests revealed multiple alterations in the fALFF **(B)** and ReHo **(D)** between each pair of groups. “*” indicates statistically different.

We failed to find any brain regions with significant differences in the value of ALFF among the three groups. However, differences in fALFF were noticed in the bilateral insula, right precentral gyrus, and left middle frontal gyrus (Table 2 and Figure 1). Compared with the non-SA and HC groups, the SA group had higher fALFF in the bilateral insula and right precentral gyrus. Meanwhile, both the SA and non-SA groups showed reduced fALFF values in the left middle frontal gyrus (MFG) compared to the HCs. In addition, significant differences in ReHo were found among the three groups in the following five regions: left MFG, left superior temporal gyrus (STG), left middle cingulate cortex (MCC) extending to the supplementary motor area (SMA), right insula, and right precentral gyrus (Table 2 and Figure 1). *Post hoc* analysis revealed that the SA group had higher ReHo in the left STG, left MCC, right insula, and right precentral gyrus, compared with the non-SA and HC groups. The SA group had lower ReHo in the left MFG compared to the HC group in addition. Compared with the HCs, the non-SA group showed lower ReHo in the left MFG and higher ReHo in the left STG.

**TABLE 2:** Significant differences in fALFF and ReHo among SA, non-SA, and HC groups.

Regions	peak MNI coordinates			Voxel size	*F/T*-value
	*x*	*y*	*z*		
**fALFF**					
**Three-group comparison**					
Right insula	39	–30	18	245	20.934
Left insula	–42	–30	15	158	20.095
Right precentral gyrus	39	–21	54	56	12.314
Left middle frontal gyrus	–27	54	12	27	14.355
**SA vs. non-SA**					
Right insula	33	–24	15	83	5.890
Left insula	–42	–33	15	123	6.045
Right precentral gyrus	36	–18	48	27	4.299
**SA vs. HC**					
Right insula	39	–27	18	167	6.015
Left insula	–39	–30	12	81	5.967
Right precentral gyrus	39	–18	39	32	4.195
Left middle frontal gyrus	–27	54	12	27	–5.409
Non-SA vs. HC					
Left middle frontal gyrus	–42	45	12	10	–3.902
ReHo					
**Three-group comparison**					
Left middle frontal gyrus	–24	51	12	18	17.195
Left superior temporal gyrus	–45	–24	6	27	13.885
Left middle cingulate cortex	0	–18	66	189	18.091
Right insula	36	–12	15	26	13.225
Right precentral gyrus	33	–33	45	73	14.277
SA vs. non-SA					
Left superior temporal gyrus	–45	–24	6	7	3.905
Left middle cingulate cortex	0	–18	66	173	5.709
Right insula	36	–12	15	18	4.195
Right precentral gyrus	30	–27	63	50	4.389
**SA vs. HC**					
Left middle frontal gyrus	–24	51	12	18	–6.020
Left superior temporal gyrus	–45	–24	6	13	4.409
Left middle cingulate cortex	3	–39	54	43	5.253
Right insula	36	–12	15	21	4.686
Right precentral gyrus	33	–24	51	27	4.296
**Non-SA vs. HC**					
Left middle frontal gyrus	–24	48	9	10	–3.611
Left superior temporal gyrus	–45	–24	6	14	3.768

fALFF, fractional amplitude of low frequency fluctuations; ReHo, regional homogeneity; MNI, Montreal neurological institute; SA, suicide attempters; non-SA, non-suicide attempters; HC, healthy controls.

### Functional connectivity analysis

**FIGURE 2:**
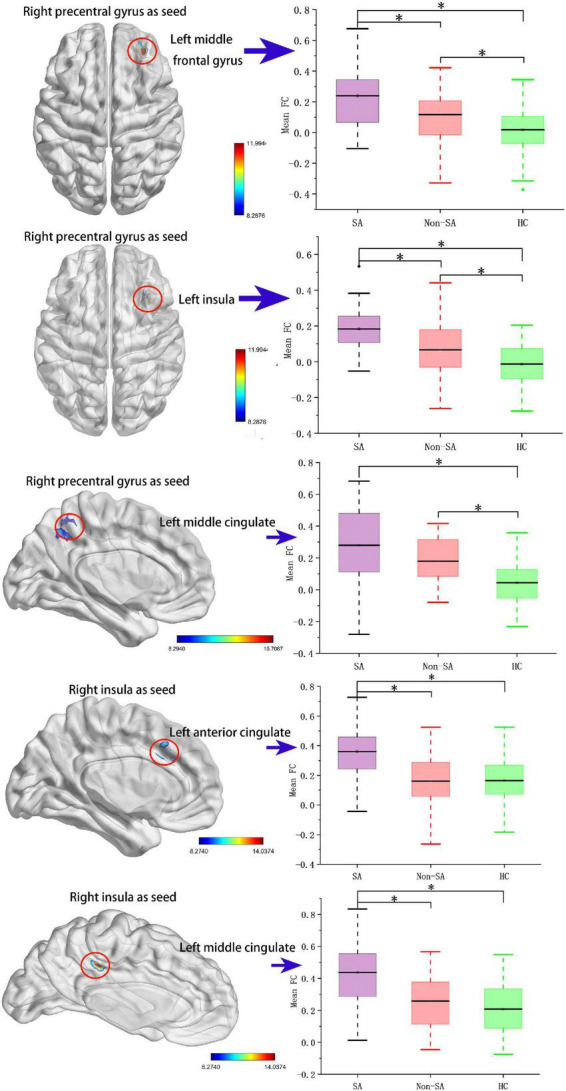
Seed-based functional connectivity analysis. The functional connectivity between the right precentral gyrus and the left middle frontal gyrus, left insula, and left middle cingulate cortex, and the functional connectivity between the right insula and left anterior cingulate cortex and left middle cingulate cortex are significant different among SA, non-SA, and HC groups, Monte Carlo simulation corrected. “*” indicates statistically different.

The following five ROIs were defined based on the regions in which fALFF/ReHo differed in the current study: the left insula (peak MNI coordinate: *x* = –42, *y* = –30, *z* = 15, voxel size: 158), right insula (peak MNI coordinate: *x* = 39, *y* = –30, *z* = 18, voxel size: 245), left MFG (peak MNI coordinate: *x* = –27, *y* = 54, *z* = 12, voxel size: 27), right precentral gyrus (peak MNI coordinate: *x* = 39, *y* = –21, *z* = 54, voxel size: 56), and left MCC (peak MNI coordinate: *x* = 0, *y* = –18, *z* = 66, voxel size: 189). FC differences were subsequently identified between the right precentral gyrus and left MFG, left insula, and left MCC, and between the right insula and left anterior cingulate cortex (ACC) and left MCC (Table 3 and Figure 2). Specifically, compared with the non-SA group, the SA group demonstrated enhanced FC between the right precentral gyrus and left MFG and left insula, and between the right insula and left ACC/MCC. Compared to HCs, the SA group showed increased FC between the right precentral gyrus and left MFG, left insula, and left MCC, and between the right insula and left ACC/MCC. The non-SA group demonstrated higher FC between the right precentral gyrus and left MFG, left insula, and left MCC than the HC group.

**TABLE 3:** Significant differences in mean FC among SA, non-SA, and HC groups.

Regions	peak MNI coordinates			Voxel size	*F*-value
	*x*	*y*	*z*		
**The right precentral gyrus as seed**					
**Three-group comparison**					
Left middle frontal gyrus	–30	45	27	102	15.365
Left insula	–36	15	6	95	15.444
Left middle cingulate cortex	3	–54	63	209	15.709
**The right Insula as seed**					
**Three-group comparison**					
Left anterior cingulate cortex	–9	27	27	186	14.037
Left middle cingulate cortex	–9	–33	42	87	15.532

FC, functional connectivity; MNI, Montreal neurological institute; SA, suicide attempters; non-SA, non-suicide attempters; HC, healthy controls.

### Correlations between functional magnetic resonance imaging metrics and clinical characteristics

**FIGURE 3:**
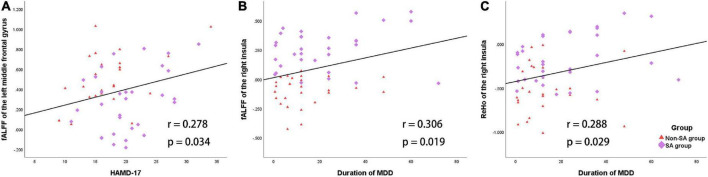
Correlations between the fALFF/ReHo and the clinical characteristics in the patient group (SA and non-SA groups). **(A)** Mean fALFF values of the left middle frontal gyrus and HAMD-17 scores were positively correlated. **(B)** Mean fALFF values of the right insula and MDD episode duration were positively correlated. **(C)** Mean ReHo values of the right insula and MDD episode duration were positively correlated.

Within the patient group (SA and non-SA combined, *n* = 58), average fALFF values of the left MFG were positively correlated with HAMD-17 scores (*r* = 0.278, *p* = 0.034, Figure 3). Additionally, mean fALFF (*r* = 0.306, *p* = 0.019) and ReHo (*r* = 0.288, *p* = 0.029) values of the right insula were positively correlated with MDD episode duration (Figure 3).

## Discussion

We applied a voxel-wise, data-driven approach to identify rs-fMRI alterations in drug-naïve, first-episode female adolescent MDD patients with and without SA. ALFF is one of the most commonly used fMRI metrics, which may reflect the intensity of regional spontaneous brain activity (9). However, fALFF has been suggested and shown to be more sensitive and specific in detecting spontaneous brain activities than ALFF (10). Similarly, ReHo is used to describe regional brain activity by calculating the similarity of the time series of a given voxel with its adjacent neighbors (27). In addition, resting-state FC has been widely used to reveal the correlations in brain activity of an ROI with discrete brain regions (29). All the above indices have been found to be valuable and reliable in various psychiatric studies. In the present study, compared with the adolescents with MDD without SA, the SA group exhibited increased fALFF in the bilateral insula and right precentral gyrus, and increased ReHo in the left STG, left MCC, right insula, and right precentral gyrus. In addition to the above changes, the SA group showed decreased fALFF and ReHo in the left MFG relative to the HCs. Moreover, additional FC analysis showed that the SA group showed increased FC between the right precentral gyrus and the left MFG and left insula, and between the right insula and ACC/MCC compared to the non-SA and HC groups. Our findings identified multiple differences within the frontolimbic circuit, which may enhance our understanding of the neuropathological basis underlying female MDD with SA during adolescence.

The present study concentrated strictly on female adolescents with MDD. Prominent sex differences in MDD have been well established (30). Patients with MDD show sex differences in terms of gene expression (31), endocrine system (32), and cortical morphometry (19). However, there are relatively few studies on sex differences in adolescent MDD. A recent study demonstrated sexually divergent development of MDD-related networks (the default mode network and limbic cortex) during healthy adolescence (22). Additionally, Whittle et al. reported that depression was correlated with increased amygdala growth in girls but decreased growth in boys in a longitudinal study of adolescent depression, and depressive symptoms were also correlated with reduced volume of nucleus accumbens only in girls (33). Taken together, it is critical that we consider gender differences in brain structure and possibly in brain function during adolescence. Therefore, research focused on females has the potential to advance our understanding of the neurobiology basis of MDD during adolescence. To the best of our knowledge, the current study is the first report on the functional alterations of drug-naïve, first-episode female adolescents with MDD.

Our study found anomalies in multiple rs-fMRI indices of the bilateral insula among the three groups. The insula has been shown to contribute to multiple functions ranging from sensorimotor, socio-emotional, and high-level cognitive functions to risky decision-making (34). Dysfunction of the insula may lead to abnormal subjective feeling states, cognitive deficits, and motivational deficits (35). Various structural and functional MRI studies reported alterations in the insular regions in MDD patients. A previous meta-analysis confirmed a smaller gray matter volume of the left insula in first episode, medication-naïve adult patients with MDD (36). Hu et al. reported decreased ALFF in bilateral insula and reduced FC between the right insula and bilateral precentral gyri in adolescents and young adults with MDD (37). Similarly, another fMRI study revealed lower degree centrality in the right insula in adolescent MDD patients (38). Furthermore, the insula may participate in the neural mechanism of suicide in MDD. A voxel-based morphometry study on late-onset depression demonstrated decreased bilateral insular volume in patients with SA compared to patients without SA (39). Another single-photon emission computed tomography study found higher regional cerebral blood flow of the right insula in depressed patients with SA (40). A recent study highlighted the resting-state FC of insular subdivisions in adolescent and young adults MDD with SA: the authors found enhanced FC of the left posterior insula with the left inferior frontal gyrus and the bilateral paracentral lobule extending to the bilateral MCC and enhanced FC of the right posterior insula with the orbital part of the left superior frontal gyrus in the SA group compared with the non-SA group (15). Partly in line with this report, we found increased FC of the MCC with the right insula in the SA group. However, we failed to find FC changes between the insula and superior/inferior frontal gyri, which may be partly explained by differences in seed choice, sex, and age ranges between the two studies. Nevertheless, the evidence from these studies provides further support for our findings implicating the insula in adolescent MDD with SA.

We found increases in fALFF/ReHo in the right precentral gyrus and in FC between the right precentral gyrus and the left insula in the SA group, when compared to both non-SA and HC groups. The precentral gyrus is part of the primary motor cortex and participates in the execution of voluntary motor movements, and previous neuroimaging studies have shown that it may be associated with MDD and suicide (15). Reduction in gray matter volume in bilateral precentral gyri in patients with MDD has been reported (41), while diminished cortical thickness has been found in the bilateral precentral gyri in young adults with suicidal ideation compared with HCs (42). Additionally, Wang et al. enrolled 18 first-episode, treatment-naive adults with MDD and found significant increases in fALFF in the right precentral gyrus, right inferior temporal gyrus, bilateral fusiform gyri, and cerebellum in the MDD group (43), which is partly in line with our results. There are relatively few studies on the precentral gyrus in adolescents with MDD. Schmaal et al. conducted a large-scale, multiple-center study, and they found a decrease in the surface area in the left precentral gyrus in adolescents with MDD, when compared with HCs (44). Mao et al. reported an elevated ReHo value in the right precentral gyrus in adolescent MDD relative to HCs (14), which is in line with our results. Further, fALFF in the right precentral gyrus in depressed adolescents with suicidal ideation was found to be significantly decreased after electroconvulsive therapy (17), and higher FC between the right precentral gyrus and left habenula was also noted in individuals with MDD or bipolar disorder and a history of SA relative to patients without SA (45). Taken together with the above studies, our findings suggest that the right precentral gyrus may be involved in the neural circuitry of suicide in MDD.

In our study, both SA and non-SA groups showed significantly lower neural activity in the left MFG than HCs. Several previous studies have demonstrated structural as well as functional alterations in the left MFG in adolescents with MDD (13, 46–48). The MFG is involved in regulating the default mode network activity *via* an antagonistic relationship, and in addition, it constitutes a core portion of the dorsolateral prefrontal cortex (DLPFC), which is the main hub of the central executive network responsible for executive and cognitive control such as response inhibition and working memory (49). By contrast, Wang et al. (50) enrolled 30 adolescents with MDD and found that patients showed elevated fALFF in the left MFG relative to HCs at baseline, and that fALFF values increased further after electroconvulsive therapy. This discrepancy in fALFF alterations may partly be related to sex differences across the patients in the two studies. Wang et al. failed to find a correlation between pretreatment fALFF values in left MFG and HAMD scores, which limited the clinical significance of elevated fALFF values. Nevertheless, further studies are needed to verify the functional alteration of the left MFG. In addition, we observed hyperactivity of the MCC-SMA in the SA group. The MCC is involved in skeletomotor control and selection based on the reward of a potential movement (51, 52), while the SMA may participate in suicidal behavior (53) as it is associated with high-order executive function related to motor planning and initiation (54). Taken together, since the aberrant frontolimbic circuit has been well elucidated in the literature, our findings provided further evidence that the frontolimbic circuit plays a critical role in the pathogenesis of adolescent MDD.

We found both SA and non-SA groups showed increased ReHo values in the left STG relative to HCs. The STG is part of the auditory network (55), and it may participate in emotion and mood regulation (56, 57). Increased neural activity in the left STG in MDD patients has been reported (58), which is in line with our results. Our finding supports that the left STG may play a role in the pathogenesis of adolescent MDD.

We note significant differences among the results of several rs-fMRI indices in our study. On the one hand, we observed functional abnormalities of the three brain regions (right insula, right prefrontal gyrus, and left MFG) across fALFF, ReHo, and FC approaches simultaneously, suggesting that these regions may be core hubs associated with the pathogenesis of female adolescent MDD. On the other hand, alterations in the left insula, left STG, and MCC among different rs-fMRI indices exhibited significant discrepancies. Whole brain ALFF values did not differ significantly among the groups, while fALFF values were statistically different in several regions. ALFF is potentially more sensitive to distinguish differences between individuals and groups, whereas fALFF may be a more specific index of low-frequency oscillations (59). Our other index, ReHo, reflects local connectivity with adjacent voxels (8). Thus, the physiological implications of fALFF and ReHo differ. Our results suggests these rs-fMRI indices may differ in sensitivity and specificity, which highlights the value of calculating multiple rs-fMRI indices to explore the functional alterations of the brain.

There are some limitations in our study. First, this was a cross-sectional study, which necessitates longitudinal studies to compare functional alterations before and after a suicide attempt. Second, we did not assess the severity of SA with scales (e.g., Columbia-Suicide Severity Rating Scale), and we did not design this initial study to examine number of suicidal attempts and suicidal methods, which may limit the interpretations of the results. We plan to collect such clinical information in more detail in future research. Finally, although we used FC to assess the correlation between brain regions, we recognize its lack of directionality in contrast to effective connectivity, which would have required us to include task-based fMRI. Further longitudinal studies with a larger sample size and task-based as well as resting-state methods are needed.

In conclusion, we provided the first report on functional alterations of drug-naïve, first-episode female adolescents with MDD. Compared with adolescents with MDD without SA, the SA group exhibited increased fALFF in the bilateral insula and right precentral gyrus, and increased ReHo in the left STG, left MCC, right insula, and right precentral gyrus. In addition to the above alterations, the SA group exhibited lower fALFF and ReHo in the left MFG relative to the HCs. Moreover, the SA group showed increased FC between the right precentral gyrus and the left MFG and left insula, and between the right insula and ACC/MCC compared to the non-SA and HC groups. In addition, the fALFF value in the left MFG was positively correlated with HAMD-17 scores, and the mean values of fALFF/ReHo in the right insula were positively correlated with MDD episode duration within the adolescents with MDD. Our findings identified multiple abnormalities of the frontolimbic circuit (especially the right insula and precentral gyrus), which may enhance our understanding of the neuropathological basis underlying female adolescents with MDD and SA.

## Data availability statement

The raw data supporting the conclusions of this article will be made available by the authors, without undue reservation.

## Ethics statement

The studies involving human participants were reviewed and approved by the Research Ethics Committee of The First Affiliated Hospital of Chongqing Medical University. Written informed consent to participate in this study was provided by the participants’ legal guardian/next of kin.

## Author contributions

ML, FL, and XZ designed the study. YH, RY, and XL participated in the patient recruitment. YH, YLi, and RY performed the MRI scanning and quality assessment. ML and YH performed the data analyses and statistics, and wrote the manuscript. XL, YLo, FL, and XZ revised it critically for important intellectual content. All authors approved the final version to be published.

## Footnotes

1
http://rfmri.org/dpabi

2
https://www.fil.ion.ucl.ac.uk/spm/software/spm12/
